# Ageist attitudes are already evident in pre‐ and early‐school children: A multi‐method examination

**DOI:** 10.1111/bjdp.70000

**Published:** 2025-06-16

**Authors:** Jenny Jaquet, Lena‐Emilia Schenker, Jennifer A. Bellingtier, Anna E. Kornadt, Michaela Riediger

**Affiliations:** ^1^ Department of Developmental Psychology Friedrich Schiller University Jena Jena Germany; ^2^ Research Institute of Child Development and Education University of Amsterdam Amsterdam The Netherlands; ^3^ Department of Behavioural and Cognitive Sciences University of Luxembourg Esch‐sur‐Alzette Luxembourg

**Keywords:** ageism, attitudes towards older adults, childhood

## Abstract

We examined age‐related attitudes in 56 German children (*M*_age_ = 6.5, 4–8 years; 55% female) using newly developed behavioural (seating and team formation task), explicit (picture rating) and implicit [single‐target implicit association test (ST‐IAT)] measures. Stimuli comprised pictures of younger and older adults. Children placed younger adults closer to themselves and placed more older adults in an opposing team, rated pictures of younger adults more positively than those of older adults, and evinced more favourable implicit evaluations of younger than older targets. This shows that already young children evaluate younger and older adults differently, underscoring the need for further research on the development of age‐related attitudes in childhood.

**Statement of Contribution**

**What is already known on this subject**

- Ageism—predominantly negative attitudes and discriminatory behaviours towards older adults—is widespread.
- Negative age attitudes can become self‐fulfilling prophecies as the individuals holding them age themselves.
- Already, children report more favourable attitudes towards younger adults compared to older adults.

**What the present study adds**

- New behavioural measures of age attitudes in pre‐ and early‐school‐aged children are developed.
- Children's preference for younger vs. older adults is evident across explicit, behavioral and implicit measures.

## INTRODUCTION

Age is a central personal characteristic used early in life to categorize people: Children as young as 4 years recognize whether a person is younger or older (Montepare & Zebrowitz, 2002). They can also describe characteristics and behaviours that they think are typical of older adults (Flamion et al., 2020; Robinson & Howatson‐Jones, 2014). Such beliefs stem from children's experiences with older adults and culturally shared age stereotypes that children are exposed to (Gilbert & Ricketts, 2008; Montepare & Zebrowitz, 2002).

The aim of the present study was to investigate age attitudes in pre‐ and early‐school‐aged children. Specifically, we were interested in whether signs of ageism towards older adults are already evident at that young age. We use the umbrella term age attitudes to refer to children's feelings, beliefs and behaviours towards older people, which can manifest on both explicit and implicit levels (Levy, 2009). Explicit attitudes are accessible to conscious awareness and can hence be reported. Implicit attitudes are present without conscious awareness and need to be inferred from people's behaviour (Eagly & Chaiken, 2007). Age attitudes are referred to as ageist when they reflect stereotypical beliefs and prejudice and predispose discriminatory behaviours towards older people (Kite & Wagner, 2002). The current study extends previous work, which primarily investigated school‐aged children with a limited range of methods. Using newly developed assessment paradigms, we employed a multi‐method approach to examine both explicit and implicit age attitudes in 4‐ to 8‐year‐old children. Below, we first highlight the relevance of understanding the development of ageist attitudes in children by elucidating their inter‐ and intrapersonal as well as their societal consequences.

### Consequences of ageist attitudes

Although culturally shared age stereotypes can encompass positive attributes like wisdom and warmth, they are rather negative overall, including beliefs that ageing leads to declines in physical functioning and cognitive problems (Hummert, 2011). This predominantly negative view of old age is widely shared among individuals and in many societies (Löckenhoff et al., 2009). It shapes what people think and feel about, and how they behave towards older people, but it also affects how they themselves age (Levy, 2009). Age attitudes thus have both intra‐ and interpersonal consequences.

At the intrapersonal level, age attitudes have been found to shape individuals' later ageing processes. Holding negative attitudes towards older people might benefit younger individuals as these attitudes valorize their own age group. However, unlike memberships in other social groups (e.g., gender), people change their age‐group membership as they grow older. At some point in life, the previously negatively evaluated outgroup of older people becomes the ingroup. Stereotype Embodiment Theory (Levy, 2009) proposes that age stereotypes acquired early in life are internalized and become self‐fulfilling prophecies as they become self‐relevant. Indeed, numerous studies have shown that individuals who held more negative age attitudes as younger adults, on average, are less healthy and satisfied in later adulthood and tend to die at a younger age compared to people with more favourable attitudes (see Wurm et al., 2017, for a review). Understanding how age attitudes develop might thus provide a lever to facilitate healthy and successful aging as today's young individuals grow older.

At the interpersonal level, age attitudes can lead to ageism, a societal problem characterized by prejudice and discriminatory actions against other people based on their age (Butler, 1980). Ageism can manifest in hostile (e.g., refusing to hire someone due to their age) and benevolent forms (i.e., patronizing views and overaccommodation towards older adults) (Cary et al., 2017). Both these forms are detrimental for older individuals (e.g., Hehman & Bugental, 2015; Shippee et al., 2019; Stokes & Moorman, 2020).

Experiences of ageism, such as in the workplace and within the healthcare system, can limit older adults' ability to actively participate in society (Shiovitz‐Ezra et al., 2018). On the societal level, ageism can thus weaken social cohesion and foster age segregation (Hagestad & Uhlenberg, 2005), but it also has economic consequences (Levy et al., 2020). The widespread acceptance of the ‘doddering but dear’ stereotype (Cuddy & Fiske, 2002) may further result in older adults being denied power or a voice in societal decision‐making processes (Swift et al., 2018).

To date, efforts to reduce ageism typically target attitudes in adults long after they have been internalized (Lytle et al., 2021; Lytle & Levy, 2019). A more effective way would be to counteract ageist attitudes before they become consolidated. For this, a better understanding of how age attitudes develop early on is essential, and the present study seeks to contribute new insights in these regards.

### The formation of age attitudes in childhood

Comparatively little research has addressed how age attitudes develop in early life. The formation of other intergroup attitudes in childhood (e.g., racial attitudes), however, has received considerable attention, and respective theories are informative for deriving hypotheses about when age attitudes manifest in children.

Developmental Intergroup Theory (DIT) (Bigler & Liben, 2007) posits that already young children use salient perceptual attributes, such as outward appearance, to categorize people into groups. This categorization process is assumed to be the basis for developing group‐related stereotypes and prejudice. Children develop attitudes towards these groups by integrating own experiences with stereotypes in their environments, such as how these groups are talked about and which attributes are assigned to them.

According to Social Identity Development Theory (SIDT) (Nesdale, 2017), most school‐aged children perceive outgroup members as similar to each other and as distinct from the ingroup. This does not necessarily mean that the outgroup is disliked, but rather liked less than the ingroup. A transition to outgroup negativity may occur after the ages of 6–7 years if, for instance, the standing of the ingroup can be enhanced by devaluing the outgroup.

These theoretical processes might also be relevant for the development of age attitudes. Age is associated with salient physical characteristics (Montepare & Zebrowitz, 2002) and can thus be easily recognized. The salience of age as a social category might be further enhanced by children's experiences that adults often label and differentiate individuals according to their age, highlighting that there might be meaningful differences between age groups. This suggests that age attitudes might evolve around the same time in life as other interpersonal attitudes, such as those regarding race or gender, namely in pre‐ and early‐school age.

The Social Reasoning Developmental Perspective (SRD) (Rutland et al., 2010) further describes children's prejudice to arise not only from group identity processes but also emphasizes their interplay with children's application of moral principles. It proposes that social judgements are also shaped by social norms and consequences for self‐representation. Ageism is often regarded as the most socially acceptable prejudice, with studies showing benevolent ageism being widely accepted (e.g., Horhota et al., 2019). Consequently, children may be less inclined to suppress explicit age attitudes, making age bias evident in childhood.

The theories presented so far relate primarily to the development of explicit attitudes. Less attention has been paid to the emergence of implicit attitudes. Available evidence suggests that implicit attitudes are acquired early in life—perhaps even earlier than explicit attitudes. Some research shows that implicit attitudes emerge rapidly in early childhood and remain relatively stable throughout life (Dunham et al., 2008). It suggests that implicit attitudes manifest as soon as children are exposed to new social groups, challenging alternative perspectives that proposed that implicit attitudes gradually emerge as children grow older (Dunham et al., 2008; Olson & Dunham, 2010).

While children recognize age as a key social marker (Montepare & Zebrowitz, 2002) and regularly interact with younger adults, they typically have limited contact with older adults apart from their grandparents (Burke, 1982). Their exposure might be rather indirect, for instance, through media, which, however, often portrays older individuals in a stereotypical manner (Robinson et al., 2007). Consequently, it is plausible that children already hold different implicit age attitudes towards younger and older adults. It is important to investigate how implicit age attitudes manifest in childhood, as these implicit biases can lead to discriminatory behaviours without the individual's conscious awareness, and even when explicit attitudes may appear positive (Levy & Banaji, 2002).

Studies suggest that children's age attitudes already tend to reflect the primarily negative image of old age that is shared societally, although they may comprise some positive aspects as well (see Mendonça et al., 2018, for a review). Further supporting the possibility that ageist attitudes may already emerge during childhood, a few studies found that children from 3 years of age preferred younger over older adults (Middlecamp & Gross, 2002; Miller et al., 1984). To date, however, most empirical evidence comes from investigations of elementary and middle‐school children. To understand the early development of age attitudes, more research examining pre‐schoolers is necessary.

Moreover, existing studies were often limited in their assessment, as they typically focused solely on children's explicit age attitudes. Studies with older children frequently used semantic differential or Likert‐type scales (e.g., Flamion et al., 2020; Lineweaver et al., 2017). Other studies that also included younger children often assessed age attitudes through picture‐based comparisons of younger and older adults (Babcock et al., 2016; Isaacs & Bearison, 1986; Seefeldt et al., 1977). Measures of implicit attitudes and their behavioural expressions have rarely been employed (Mendonça et al., 2018). However, the assessment of implicit, in addition to explicit, attitudes is important because children may be unaware of their attitudes or unable to express them. In addition, as children get older, they increasingly learn to tailor their explicit expressions of attitudes according to social norms (Olson & Dunham, 2010).

Only a few studies have investigated age attitudes using both implicit and explicit measurement approaches. There are two notable exceptions: Isaacs and Bearison (1986) assessed age attitudes in 4‐ to 8‐year‐old children using pictorial stimuli (assessing explicit attitudes) and behavioural measures, such as proxemic distance to older versus younger confederates during a cooperative task (assessing implicit attitudes). They found evidence for age‐based prejudice and stronger preferences for younger individuals for both explicit and implicit attitudes. More recently, Babcock et al. (2016) developed the ‘Child‐Age IAT’ to examine children's implicit age attitudes and measured explicit age attitudes using picture ratings. In a sample of elementary school children, they found significant implicit but not explicit bias, with no correlation between both measures. At this age, children may already have learned to suppress the explicit expression of age stereotypes, which are, however, evident in implicit measures. While both studies used different operationalizations, to the best of our knowledge, there is no study that examined age‐related attitudes using a combination of explicit, implicit and behavioural measures.

### The current study

This study examined age attitudes in preschool and early‐school children, addressing an existing research gap. Based on the theoretical considerations and empirical evidence outlined above, we hypothesized that already 4‐ to 8‐year‐old children exhibit less favourable explicit and implicit evaluations of older than younger adults. This age range is particularly informative, as it might mark the period in which group preferences start to emerge, but in which also first signs of outgroup negativity become evident (Nesdale, 2017). We employed a multi‐method approach, assessing children's explicit and implicit age attitudes using newly developed behavioural paradigms, an implicit association test and an explicit picture rating. This allowed us to investigate our hypothesis with regard to both explicit and implicit age attitudes.

## METHOD

### Participants and procedure

The study was conducted in Germany, which is comparable to other European and Western countries (e.g., the US) regarding attitudes towards different age groups (Weiss & Zhang, 2020) and perceived levels of age discrimination (Abrams et al., 2011).

Fifty‐six children (4–8 years, *M*_age_ = 6.5, SD = 1.58, 55% girls, 45% boys) were recruited via flyers and newspaper announcements in Jena, a university town in Germany. An a priori power analysis (paired‐sample *t*‐test, *α* = .05, power = 0.95, *d* = 0.5) indicated a required sample size of 54 individuals to demonstrate a medium‐sized difference between children's attitudes towards younger versus older adults.

All children were native German speakers. No information on racial background was assessed, which is typical in German research settings, where the vast majority of the population is White, and asking questions about one's racial background is considered discriminatory. All children were accompanied by one parent (22–50 years, *M*_age_ = 36.95, SD = 5.49, 86% mothers, 14% fathers, 54% held a university degree (incl. Doctoral degrees); see Data S1 for details).

Data collection began in November 2019 and continued until March 2020 (41 participants), when the study had to be suspended due to contact restrictions deriving from the COVID‐19 pandemic. Following the approval of hygiene protocols, we resumed testing in July 2020 (15 participants). There were no significant differences in our outcome variables between pre‐ and post‐break participant groups (see Data S1). The study was approved by the ethics committee of the Friedrich Schiller University Jena.

Upon arrival in the lab, the study procedure was explained to the children and parents. After confirming informed consent (children provided verbal, parents written consent), the child was taken to an adjoining testing room where they completed a series of ‘games’ (described below). Children first participated in two behavioural tasks (‘party game’ and ‘treasure hunt’), followed by the implicit measure (ST‐IAT), and finally, the explicit measure (picture rating task). Meanwhile, parents completed various questionnaires (e.g., on socio‐demographic information). The procedure took about an hour to complete.

### Measures

We used four measures to assess children's age attitudes: The ‘party game’ and the ‘treasure hunt’ were developed for this study as age‐appropriate behavioural indicators of age attitudes. The ST‐IAT was adapted as an implicit measure, and a picture rating task served as an explicit measure of children's evaluations of younger and older adults. In addition to age attitudes, we also assessed children's age awareness with two tasks.

#### Seating assignment task ‘party game’

**FIGURE 1:**
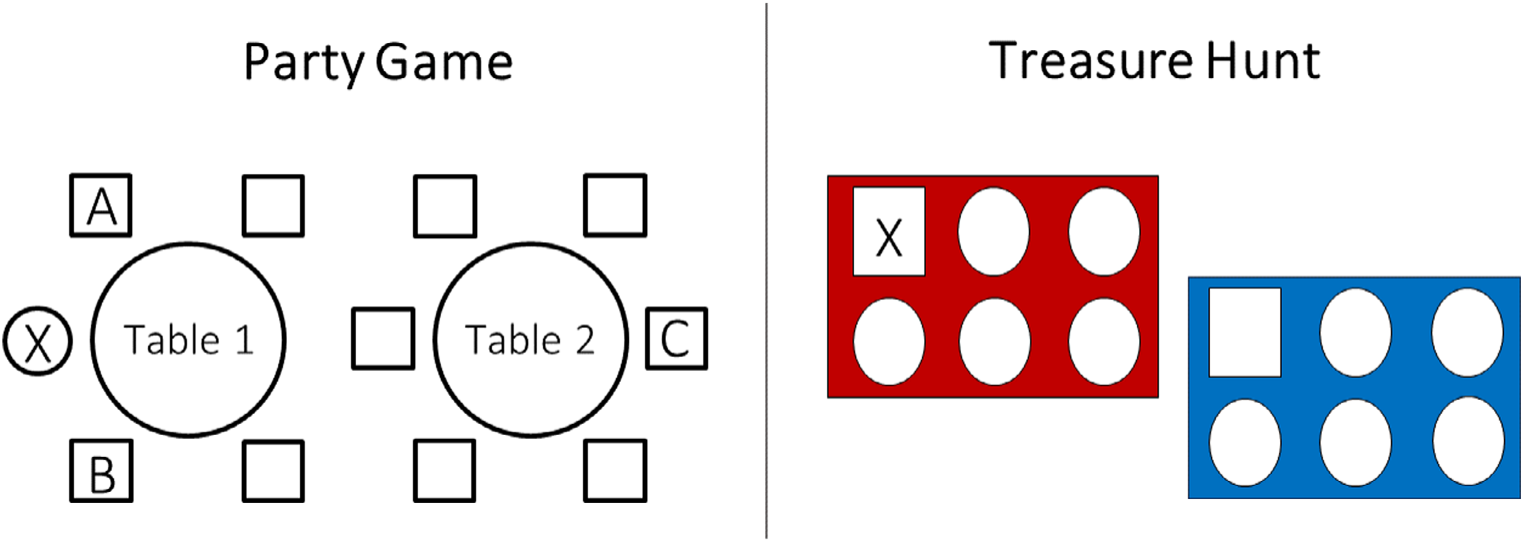
Illustrations of the party game (left panel) and the treasure hunt (right panel) paradigms. The ‘X’ marks the child's position. ‘A’ and ‘B’ mark the seats closest to the child. ‘C’ represents the seat farthest away from the child.

Children were asked to imagine they would celebrate a party. The research assistant showed them an image of a round party table with five chairs. The children were asked to place a drawn picture of themselves on the chair directly in front of them. The research assistant then placed an additional table with six seats behind the first table (see Figure 1). Next, the children were shown images of 10 individuals. The research assistant explained that these were the guests attending the party. The images were natural face photographs displaying happy expressions from the 10K faces database (Bainbridge et al., 2013). All guests were of the same sex as the child. Five guests were older (60+ years), and five were younger adults (20–30 years). The order of displaying the images alternated older and younger faces and was held constant across all participants.

The children were asked to select a guest they would like to sit next to and to place them in one of the seats next to their image (see Figure 1). They were then asked who should sit in the other seat next to them. The children were then prompted to fill the remaining seats at their table. For the second table, children were asked to first choose the person who should sit the farthest away from them and then fill in the rest. In line with proxemics research showing that social attitudes are reflected in preferred interpersonal distances (e.g., Dotsch & Wigboldus, 2008; Mehrabian, 1969), we recorded the distance (number of seats) of each party guest from the child and used the average distance to younger and older party guests as a proxy for children's attitudes towards younger and older adults.

#### Teammate selection task ‘treasure hunt’

For this task, the research assistant explained that the party guests would participate in a treasure hunt in two teams (red and blue). The same images as in the party game were used to depict the guests, and two coloured sheets were placed in front of the children. The children chose which team they wanted to be part of, and their drawn image of themselves was placed on the corresponding sheet (see Figure 1). They were then asked to assign each party guest to either their own or the opposing team. The team membership of each guest was recorded as a proxy for children's interpersonal attitudes, assuming they place people they prefer on their own team.

#### Single‐target implicit association test

Implicit attitudes were measured using an ST‐IAT (Bluemke & Friese, 2008; Wigboldus et al., 2004). This paradigm assesses the relative strength of associations between a target category (here, younger or older adult) and evaluative attributes (here, positive vs. negative; Greenwald et al., 1998). Target stimuli were the faces of four younger and four older adults (male and female faces) displaying neutral expressions. All images were retrieved from the FACES database (Ebner et al., 2010). Four happy and four sad emoji were used as stimuli to represent positive and negative evaluations. The children were asked to sort the target images and the attribute emoji to either the green (left) or blue (right) side of the screen. Both sides were marked with symbols of the corresponding categories.

Two practice trials were conducted in which children first sorted targets and attributes separately, one involving the sorting of younger and older faces and the other of happy and sad emoji to the respectively assigned sides of the screen. During these practice trials, feedback was provided via thumbs‐up or thumbs‐down images. In the test trials, children were presented, one by one, with both faces and emoji and asked to sort these to the corresponding sides of the screen. Throughout all test trials, both categories of evaluative stimuli were presented, and their assignment to response sides did not alter (i.e., happy emoji were always assigned to the left, sad emoji to the right side). Only one category of target stimuli (young or old) was presented per test trial, and the correct response (assignment to the left or right) was varied. The test trials thus included four conditions that were presented in randomized order and varied in the combination of target and attribute categories sharing the same response side (i.e., positive + old, negative + old, positive + young, negative + young), with the respectively remaining attribute category (i.e., negative or positive) being assigned to the other response side. Children's response times for completing the assignment tasks were measured. The basic assumption of IAT paradigms is that faster reaction times indicate stronger implicit associations of the concepts that share the same response options. We thus computed the children's mean reaction times for each of the combinations of target and attribute categories as well as *D* scores for younger and older targets (see Greenwald et al., 2003). A more positive *D* score represents a higher association of the respective group with positive attributes.

#### Picture rating task

The picture rating task included the evaluation of all face images of younger and older adults from both the party game and the IAT task using a 5‐point smiley‐face Likert scale called the ‘Five Degrees of Happiness Scale’. Response options include smileys with varying intensities of happiness expressions (see Hall et al., 2016). Children first practiced the usage of the scale with neutral stimulus material. They were then asked to evaluate the images of younger and older adults (‘How much do you like this person?’). The least happy face was coded with a 1, and the happiest face with a 5. Ratings were averaged across all younger and all older face images, respectively, deriving scale scores of children's evaluations of younger (α = .81) and older faces (α = .89).

#### Indicators of children's age awareness

To assess whether children were able to distinguish between older and younger persons, we obtained two indicators of age awareness. First, the children were asked to indicate which of the party guests they would consider as old. Children were considered age‐aware if they committed no more than one error (i.e., not identifying an older adult as old or identifying a younger adult as old). Most children (*n* = 48) successfully completed this task. The seven children who were unable to complete this task were 4–6 years old, with the majority of them being 4 years old (*n* = 4). For one child, information on this task was missing. The seven children who could not solve the task were shown the correct answer before proceeding.

As a second indicator of age awareness, children were asked to sort the ST‐IAT target faces according to their age. All children who successfully completed the first age awareness task also correctly sorted the ST‐IAT target faces. Of the seven children who did not successfully solve the first age awareness task, two completed the second age awareness task. Five children were unable to complete both age awareness tasks correctly.

We conducted all analyses, including and excluding the children who failed the age awareness tasks. The pattern of results remained consistent. In the interest of parsimony, we only report findings from the complete sample.

## RESULTS

The data and code necessary to reproduce the analyses are available at https://osf.io/crkgn/.

### Missing values and treatment of outliers

One IAT value was missing due to technical malfunctions during testing. Values with a *z*‐score > 3.29 or <−3.29 were considered outliers (Tabachnick & Fidell, 2013, p. 107) and winsorized. In line with previous research (Cvencek et al., 2011), we excluded ST‐IAT scores of 9 participants who responded faster than or equal to 300 ms in 10% of their responses and whose error rate was larger than or equal to 35%, which suggests careless responding.

### Children's age attitudes

Table 1 displays means, standard deviations and bivariate correlations between the age attitude measures. We found positive correlations among the behavioural measures, which were also linked to explicit ratings of older adults. Explicit and implicit measures showed no correlation. While explicit ratings of both age groups were positively associated, no correlation was observed between the two *D* scores.

**TABLE 1:** Means, standard deviations and Pearson correlation between age attitudes measures.

Variables	*M* (SD)	1	2	3	4	5	6	7	8	9	10	11
Party game												
1. Mean distance OAs	2.78 (0.82)	–										
2. Mean distance YAs	1.82 (0.82)	−1.00**	–									
Treasure hunt												
3. Sum OAs opposing team	3.36 (1.37)	.69**	−.69**	–								
4. Sum YAs opposing team	1.63 (1.37)	−.68**	.68**	−1.00**	–							
Picture rating												
5. Mean picture rating OAs	2.52 (0.91)	−.45**	.45**	−.34*	.33*	–						
6. Mean picture rating YAs	3.17 (0.79)	.10	−.09	.13	−.12	.45**	–					
ST‐IAT												
7. *D* score OAs	−0.02 (0.45)	.25	−.25	.09	−.08	−.06	−.14	–				
8. *D* score YAs	0.21 (0.44)	−.06	.06	−.04	.06	.24	.22	−.01	–			
9. Mean RT OAs + positive	1.63 (0.58)	−.22	.22	−.24	.19	.16	−.13	−.41**	−.05	–		
10. Mean RT OAs + negative	1.60 (0.53)	−.09	.10	−.19	.15	.12	−.23	.24	−.10	.75**	–	
11. Mean RT YAs + positive	1.55 (0.48)	−.04	.04	−.21	.19	−.05	−.10	.00	−.35*	.69**	.72**	–
12. Mean RT YAs + negative	1.71 (0.58)	−.05	.06	−.22	.22	.05	.03	−.09	.37*	.70**	.66**	.69**

Abbreviations: Negative and positive,type of evaluative stimuli presented together with the respective target category (young vs. old); OAs, older adults; RT, reaction time (in seconds); YAs, younger adults.

*p < .05; **p < .01.

Wilcoxon‐signed rank tests indicated that the mean physical distance children assigned to party guests in the party game was significantly smaller for younger adults (*Mdn* = 1.80) than for older adults (*Mdn* = 2.90), *T* = 328.5, *z* = −3.84, *p* < .001, *r* = −.51 and that children placed significantly more older (*Mdn* = 4.00) than younger adults (*Mdn* = 1.00) on the opposing team in the treasure hunt game, *T* = 304.50, *z* = −4.08, *p* < .001, *r* = −.55. Similarly, a Wilcoxon‐signed rank test also indicated that children rated pictures of younger adults significantly more positively (*Mdn* = 3.21) than pictures of older adults (*Mdn* = 2.57), *T* = 1279.0, *z* = 4.99, *p* < .001, *r* = .67.

Results from the ST‐IAT showed that, on average, children's older adult *D* scores (*M* = −0.02, SE = 0.07) were smaller than children's younger adult *D* scores (*M* = 0.21, SE = 0.07). A paired‐sample *t*‐test showed that this difference, 0.23, was significant, *t*(45) = 2.41, *p* = .020, and represented an effect of *d* = 0.36. We found no evidence that children's reaction times for categorizing older adults differed depending on whether the categorization shared the same response as categorizing the attribute ‘negative’ versus ‘positive’ emoji: A paired‐sample *t*‐test indicated that this difference, −0.04 s, was not significant, *t*(45) = −0.63, *p* = .531, and represented an effect of *d* = −0.09. On average, however, children categorized younger targets more slowly when this categorization shared the same response as categorizations of negative emoji (*M* = 1.71 s, SE = 0.09 s) compared to when the response was shared with positive emoji (*M* = 1.55 s, SE = .07 s). This difference, 0.16 s, was significant, *t*(45) = 2.51, *p* = .016, and represented an effect of *d* = 0.37.

## DISCUSSION

Our findings provide consistent evidence that the children in our study preferred younger over older adults: They placed younger adults closer to themselves in the party game and placed more older adults in the opposing team in the treasure hunt game. They also rated younger adults more positively than older adults in an explicit picture rating task. Also, the ST‐IAT indicated that the children showed more favourable implicit attitudes towards younger than towards older adults. In line with DIT (Bigler & Liben, 2007), children seem to use age as a salient social category to which they attach meaning, as evidenced by their differential behaviour towards and evaluations of younger and older adults.

Evidence was less clear for a potential devaluation of older people. Although children in our study preferred to be closer to younger people in the behavioural measures, they did not show a negative attitude towards older adults, neither in the picture rating task nor in the ST‐IAT, where there was no evidence for a significant difference in the association strengths of older adults with positive compared to negative emoji. As SIDT (Nesdale, 2017) suggests, outgroup devaluation is more likely when a group is perceived as a threat or when it benefits the ingroup's status. For the social group of older adults, at least in childhood, this may not apply. Children might have very little contact with older people other than their grandparents, so this might also be a group that they have little personal experience with.

Taken together, these findings show that already children at preschool to early‐school age have acquired ageist attitudes in the sense of favouring younger over older adults, albeit they do not yet display signs of dislike of older adults. This pattern of results largely converged across a range of different implicit and explicit, behavioural as well as evaluative measures, evidencing the stability of our findings.

However, we found neither significant associations between explicit and implicit measures nor between behavioural and implicit measures. One reason for this finding could be that our sample size—determined for comparing children's attitudes towards younger versus older adults—was insufficient for investigating associations between measures. Thus, the following substantive interpretations should be considered preliminary.

Substantively, the lack of correlation between explicit and implicit age attitudes aligns with previous research (Babcock et al., 2016) and might reflect differential developmental trajectories (Olson & Dunham, 2010). While explicit attitudes tend to increase until early‐school age and decline afterward (Aboud, 2008), implicit attitudes are thought to develop early and remain stable, being less susceptible to, for example, social desirability concerns (compared to explicit attitudes).

Missing links between behavioural and implicit measures might be due to our behavioural tasks reflecting indirectly measured explicit rather than implicit attitudes. Asking children to seat or choose targets likely activates controlled decision‐making and does not meet criteria for implicit measures such as unawareness, unintentionality and low cognitive demand (Olson & Dunham, 2010). Ecologically valid studies are needed to investigate how implicit and explicit age attitudes predict behaviours over time and outside of controlled laboratory settings.

Zero correlations between the implicit measures for younger and older adults might have emerged because children have distinctive associative patterns for each of the two age groups. This would mean that having a positive association with one age group does not necessarily imply a negative or less positive view of the other.

Last, it should be noted that few, mostly younger, children struggled to distinguish younger and older adults. However, as found in previous studies, the majority of 4‐year‐old children were capable of accurately sorting images (e.g., Kogan et al., 1961), suggesting heterogeneity in young children's age discriminability. This might also contribute to variability in the emergence of age attitudes in childhood.

### Limitations

Our study comes with limitations that should be addressed by future research. First, although our results are consistent with our hypothesis and align with existing empirical evidence, future research should determine the generalizability of our findings, which might be limited by the nature of our convenience sample of children from mostly highly educated family backgrounds. Although confirming our hypothesis in our relatively small sample suggests a robust effect that is likely replicable in larger studies, future research should employ our measures with larger and more diverse samples to substantiate our findings.

Furthermore, our cross‐sectional design does not provide information about the emergence and within‐person development of age attitudes in young children. Future longitudinal studies are required to better understand the developmental dynamics of emerging intergroup attitudes in childhood.

While our pictorial stimuli allowed us to investigate pre‐literate children, a limitation of pictorial measures is that there may be other target characteristics that might bias children's reactions (e.g., Montepare & Zebrowitz, 2002). Future research is needed to evaluate and control for the influence of such potentially confounding effects. Researchers should also consider varying pictorial stimuli across measures. In our study, correlations between measures may partly stem from the repeated use of the same images across tasks.

Further, the IAT procedure did not produce valid scores for nine participants (16%) due to lacking abilities or inappropriate responses. This is a slightly higher rate than reported in comparable studies (e.g., 14%, Cvencek et al., 2011). Similar to the age awareness task, the majority of these participants were 4‐year‐olds, highlighting the methodological challenges of assessing implicit bias in young children (Olson & Dunham, 2010). Although we followed recommendations for adapting IAT tasks (Cvencek et al., 2011), further modifications may be necessary. Future multi‐method studies could consider administering the IAT before other measures. In our study, children had already participated in about 20 min of testing before completing the IAT. This might have diminished cognitive resources, reducing concentration, particularly among the youngest participants. In general, the development of implicit measures to study the development of implicit social cognition in preschool children remains a challenge.

Finally, to minimize participant burden, we only investigated age attitudes on a positive–negative continuum. In order to gain a more nuanced understanding of children's age attitudes, future studies should use a multidimensional approach by considering, for example, the stereotype content dimensions warmth and competence (Vauclair et al., 2018).

## CONCLUSION

Our findings demonstrate that already children in pre‐ and early‐school age evaluate younger and older adults differently in that they prefer younger over older adults. There was no evidence that children disliked older adults. However, theories suggest that children's preference for younger over older adults could be the precursor of internalizing negative attitudes towards older adults. Introducing children to realistic and diverse information about older adults already at that young age should thus be important to prevent the internalization of negative age stereotypes. Longitudinal investigations will be needed to better understand the development of age attitudes and to explore factors (e.g., parental attitudes) that affect their formation in childhood.

## AUTHOR CONTRIBUTIONS

**Jenny Jaquet:** Investigation; data curation; formal analysis; methodology; visualization; writing – original draft; writing – review and editing. **Lena‐Emilia Schenker:** Investigation; formal analysis; writing – original draft; writing – review and editing. **Jennifer A. Bellingtier:** Conceptualization; funding acquisition; methodology; project administration; writing – review and editing; supervision. **Anna E. Kornadt:** Conceptualization; writing – original draft; writing – review and editing. **Michaela Riediger:** Conceptualization; resources; supervision; writing – original draft; writing – review and editing.

## CONFLICT OF INTEREST STATEMENT

The authors have no conflicts of interest to disclose.

## Supporting information


Data S1:

## Data Availability

The data and code necessary to reproduce the analyses presented here are publicly accessible. Data, code and all study materials that can be shared by the authors are available at https://osf.io/crkgn/. Note that some of the study materials cannot be shared due to licensing restrictions.
