# Supplementary material for: Ageist attitudes are already evident in pre‐ and early‐school children: A multi‐method examination
Source: Br J Dev Psychol. 2025 Jun 16;43(4):1027–40. doi: 10.1111/bjdp.70000 (PMC12505827; doi:10.1111/bjdp.70000)
Supplement: Supplementary file 1 — Data S1: [file BJDP-43-1027-s001.pdf]

Supporting Information A: Results for Different Participant Ages

Table A1

Study Variable Descriptive Statistics for Different Participant Ages

Variable	Age group								
	4- & 5-year olds			6-year olds			7- & 8-year olds		
	M	*SD*	Range	*M*	*SD*	Range	*M*	*SD*	Range
Party game									
Mean distance OAs	2.43	0.65	1.20 – 3.40	2.92	0.98	.80 – 3.80	2.97	0.72	1.60 – 3.80
Mean distance YAs	2.18	0.64	1.20 – 3.40	1.68	0.98	.80 – 3.80	1.63	0.72	0.80 – 3.00
Treasure hunt									
Sum OAs opposing team	3.00	1.28	0.00 – 5.00	3.61	1.58	0.00 – 5.00	3.45	1.23	0.00 – 5.00
Sum YAs opposing team	1.94	1.30	0.00 – 5.00	1.39	1.58	0.00 – 5.00	1.55	1.23	0.00 – 5.00
Picture Rating									
Mean picture rating OAs	2.76	0.76	1.00 – 4.43	2.30	1.05	1.00 – 4.00	2.49	0.89	1.00 – 4.00
Mean picture rating YAs	3.18	0.85	1.29 – 5.00	3.16	0.84	1.36 – 4.43	3.16	0.72	1.43 – 4.21
Single-Target-IAT									
D-Score OAs	0.04	0.49	-0.88 - 0.87	-0.17	0.51	-0.88 – 0.63	0.08	0.36	-0.55 – 0.82
D-Score YAs	0.24	0.52	-0.87 – 1.07	0.24	0.39	-0.43 – 0.87	0.17	0.46	-0.58 – 1.16
Mean RT OAs + negative	2.16	0.46	1.49 – 2.93	1.59	0.46	1.01 – 2.55	1.28	0.33	0.89 – 2.22
Mean RT OAs + positive	2.15	0.51	1.44 – 3.06	1.74	0.58	1.11 – 3.27	1.24	0.29	0.91 – 1.91
Mean RT YAs + negative	2.34	0.50	1.62 – 3.25	1.78	0.48	1.20 – 2.87	1.29	0.25	0.82 – 1.82
Mean RT YAs + positive	2.08	0.47	1.44 – 3.16	1.58	0.36	1.00 – 2.33	1.23	0.28	0.81 – 1.80

Note. ‘OAs’ stands for older adult, ‘YAs’ stands for younger adult, ‘RT’ stands for reaction time (in seconds).

Table A2

Wilcoxon Signed-Rank Test Results for Party Game, Treasure Hunt, and Picture Rating Scores for Different Participant Ages

Variable	Age group											
	4- & 5-year olds (n =18)				6-year olds (n =18)				7- and 8-year olds (n =20)			
	T	z	p	r	T	z	p	r	T	z	p	r
Party Game	58.5	-1.18	.237	-.28	34.0	-2.26	.024	-.53	23.0	-3.07	.002	-.69
Treasure Hunt	46.5	-1.75	.081	-.41	29.5	-2.48	.013	-.58	33.5	-2.74	.006	-.61
Picture Ratings	125.5	2.32	.020	.55	151.0	2.86	.004	.67	167.5	3.57	<.001	.80

Note. For all measures, differences were computed by subtracting older adults' scores from younger adults' scores. T = test statistic, z = standardized test statistic. For the party game, negative z-scores represent more favorable evaluations of younger adults. For the picture rating, positive z-scores represent more favorable evaluations of younger adults.

Table A3

Paired Samples T-test Results for Differences in D-scores, Older Adult Reaction Time and Younger Adult Reaction Times for Different Participant Ages

Variable	Age group														
	4- & 5-year olds (n = 11)					6-year olds (n = 16)					7- and 8-year olds (n = 19)				
	Mean diff.	t	df	p	d	Mean diff.	t	df	p	d	Mean diff.	t	df	p	d
D-scores	0.20	0.84	10	.420	0.25	0.41	2.48	15	.026	0.62	0.09	0.77	18	.453	0.18
RT OAs	< 0.01	0.03	10	.978	0.01	-0.15	-1.35	15	.198	-0.34	0.04	0.71	18	.486	0.16
RT YAs	0.27	1.28	10	.229	0.39	0.20	2.25	15	.040	0.56	0.06	1.00	18	.329	0.23

Note. OAs = older adults, YAs = younger adults, RT = reaction time in seconds. 'Mean diff' stands for mean difference. For d-scores 'Mean diff' is the difference between the d-score for younger adults and the d-score for older adults (d-score_{young} – d-score_{old}). For RT 'Mean diff' is the difference between RT when pairing the target stimulus with negative evaluative stimuli versus with positive evaluative stimuli ($RT_{negative} – RT_{positive}$).

Supporting Information B: Results for Male versus Female Children

Table B1

Study Variable Descriptive Statistics for Male and Female Children

Variable	Gender					
	Male			Female		
	M	*SD*	Range	*M*	*SD*	Range
Party game						
Mean distance OAs	2.43	0.85	0.80 – 3.60	3.06	0.67	1.40 – 3.80
Mean distance YAs	2.18	0.85	1.00 – 3.80	1.54	0.67	0.80 – 3.20
Treasure hunt						
Sum OAs opposing team	2.84	1.52	0.00 – 5.00	3.77	1.09	2.00 – 5.00
Sum YAs opposing team	2.16	1.52	0.00 – 5.00	1.19	1.08	0.00 – 3.00
Picture Rating						
Mean picture rating OAs	2.45	0.84	1.00 – 3.71	2.56	0.97	1.00 – 4.43
Mean picture rating YAs	2.96	0.87	1.29 – 5.00	3.33	0.69	2.14 – 4.71
Single-Target-IAT						
D-Score OAs	0.05	0.44	-0.71 – 0.87	-0.07	0.47	-0.88 – 0.82
D-Score YAs	0.21	0.51	-0.87 – 1.07	0.20	0.40	-0.44 – 1.16
Mean RT OAs + negative	1.53	0.52	0.89 – 2.93	1.65	0.54	0.98 – 2.67
Mean RT OAs + positive	1.47	0.35	0.92 – 2.20	1.76	0.69	0.91 – 3.27
Mean RT YAs + negative	1.62	0.53	1.00 – 2.64	1.78	0.61	0.82 – 3.25
Mean RT YAs + positive	1.48	0.50	0.98 – 3.16	1.61	0.47	0.81 – 2.44

Note. ‘OAs’ stands for older adult, ‘YAs’ stands for younger adult, ‘RT’ stands for reaction time (in seconds).

Table B2

Wilcoxon Signed-Rank Test Results for Differences in Party Game, Treasure Hunt, and Picture Rating Scores for Male Versus Female Children

Variable	Gender							
	Male (n = 25)				Female (n = 31)			
	T	z	p	r	T	z	p	r
Party Game	132.5	-0.81	.419	-.16	34.0	-4.21	<.001	-.76
Treasure Hunt	119.0	-1.19	.234	-.24	30.0	-4.33	<.001	-.77
Picture Ratings	283.0	3.25	<.001	.65	370.0	3.81	<.001	.68

Note. For all measures, differences were computed by subtracting older adults' scores from younger adults' scores. T = test statistic, z = standardized test statistic. For the party game, negative z-scores represent more favorable evaluations of younger adults. For the picture rating, positive z-scores represent more favorable evaluations of younger adults.

Table B3

Paired Samples T-test Results for Differences in D-scores, Older Adults Reaction Time and Younger Adults Reaction Times for Male and Female Children

Variable	Gender									
	Male (n = 20)					Female (n = 26)				
	Mean diff.	*t*	*df*	*p*	*d*	Mean diff.	*t*	*df*	*p*	*d*
D-scores	0.17	1.07	19	.300	0.24	0.27	2.35	25	.027	0.46
RT OAs	0.06	0.76	19	.455	0.17	-0.11	-1.29	25	.210	0.25
RT YAs	0.15	1.22	19	.238	0.27	0.17	2.55	25	.017	0.50

Note. OAs = older adults, YAs = younger adults, RT = reaction time in seconds. 'Mean diff' stands for mean difference. For d-scores 'Mean diff' is the difference between the d-score for younger adults and the d-score for older adults (d-score_{young} – d-score_{old}). For RT 'Mean diff' is the difference between RT when pairing the target stimulus with negative evaluative stimuli versus with positive evaluative stimuli ($RT_{negative}$ – $RT_{positive}$).

Supporting Information C: Results for Children Tested Before vs. During the Covid-19 Pandemic

Table C1

Study Variable Descriptive Statistics for Children Tested Before Versus During the Covid-19 Pandemic

Variable	Pandemic status					
	Before the Pandemic (n = 41)			During the Pandemic (n = 15)		
	M	*SD*	Range	*M*	*SD*	*Range*
Party game						
Mean distance OAs	2.80	0.81	0.80 – 3.80	2.73	0.85	1.40 – 3.80
Mean distance YAs	1.80	0.81	0.80 – 3.80	1.87	0.85	0.80 – 3.20
Treasure hunt						
Sum OAs opposing team	3.44	1.32	0.00 – 5.00	3.13	1.51	0.00 – 5.00
Sum YAs opposing team	1.56	1.32	0.00 – 5.00	1.80	1.52	0.00 – 5.00
Picture Rating						
Mean picture rating OAs	2.56	0.84	1.00 – 4.07	2.40	1.09	1.00 – 4.43
Mean picture rating YAs	3.14	0.75	1.29 – 5.00	3.25	0.91	1.36 – 4.47
Single-Target-IAT						
D-Score OAs	0.01	0.46	-0.88 - 0.87	-0.13	0.42	-0.71 - 0.57
D-Score YAs	0.26	0.46	-0.87 – 1.16	0.03	0.34	-0.44 - 0.49
Mean RT OAs + negative	1.52	0.48	0.89 – 2.55	1.86	0.62	1.05 – 2.93
Mean RT OAs + positive	1.53	0.50	0.91 – 2.77	1.98	0.73	1.12 – 3.27
Mean RT YAs + negative	1.66	0.56	0.82 – 3.25	1.90	0.63	1.17 – 2.87
Mean RT YAs + positive	1.48	0.50	0.81 – 3.16	1.80	0.34	1.21 – 2.33

Note. 'OAs' stands for older adults, 'YAs' stands for younger adults, 'RT' stands for reaction time.

Table C2

Mann-Whitney U Test Results for Comparing Party Game Scores Between Children Tested Before Versus During Covid-19 Pandemic

Variable	*U*	*z*	*p*	*r*
Mean distance OAs	292.00	-0.29	.773	-.04
Mean distance YAs	322.50	0.28	.780	.04

Note. *U* = test statistic, *z* = standardized test statistic, 'OAs' stands for older adult, 'YAs' stands for younger adult, n_{before} = 41 children, n_{during} = 15 children.

Table C3

Mann-Whitney U Test Results for Comparing Treasure Hunt Scores Between Children Tested Before Versus During Covid-19 Pandemic

Variable	*U*	*z*	*p*	*r*
Sum OAs opposing team	271.50	-0.69	.493	-.09
Sum YAs opposing team	332.00	0.47	.640	.06

Note. *U* = test statistic, *z* = standardized test statistic, 'OAs' stands for older adult, 'YAs' stands for younger adult, n_{before} = 41 children, n_{during} = 15 children.

Table C4

Mann-Whitney U Test Results for Comparing Picture Rating Scores Between Children Tested Before Versus During Covid-19 Pandemic

Variable	U	z	p	r
Mean picture rating OAs	281.00	-0.49	.623	-.07
Mean picture rating YAs	327.00	0.36	.718	.05

Note. U = test statistic, z = standardized test statistic, 'OAs' stands for older adult, 'YAs' stands for younger adult, n_{before} = 41 children, n_{during} = 15 children.

Table C5

Independent Samples T-test Results for Comparing D-scores and Reaction Times Between Children Tested Before Versus During Covid-19 Pandemic

Variable	Mean Diff.	*t*	*df*	*p*	*d*
D-Score OAs	0.14	0.86	44	.392	0.26
D-Score YAs	0.22	1.41	44	.166	0.43
Mean RT OAs + negative	-0.34	-1.85	44	.071	-0,56
Mean RT OAs + positive	-0.45	-2.25	44	.030	-0.68
Mean RT YAs + negative	-0.24	-1.18	44	.246	-0.36
Mean RT YAs + positive	-0.31	-1.87	44	.069	-0.56

Note. U = test statistic, z = standardized test statistic, 'OAs' stands for older adult, 'YAs' stands for younger adult, n_{before} = 36 children, n_{during} = 10 children.

Supporting Information D: Information on the Accompanying Parent

Sociodemographics

N = 56 parents participated in the study. Participating parents were aged between 22 and 50 (*M* = 37, *SD* = 5.5), the majority of which (86%) were female. Forty-nine parents indicated the age of their partner: they were aged between 24 and 51 (*M* = 38.5, *SD* = 5.9).

The majority of participating parents held a university degree (48%). The rest of the participants held the “Abitur” (German High School degree, 21%), had completed vocational training (16%), held a “Realschulabschluss” (secondary school certificate, 9%), or had a doctoral degree (5%).

Most participating parents were employed (57%) or self-employed (9%). The rest were homemakers (7%), students (7%), unemployed (4%), or not employed for opposing reasons such as being on maternity leave, in vocational training, or in public service (16%).

Older Adult Contact

Using a 5 -point scale ranging from 0 “never” to 6 “every day”**,** parents reported that they regularly see (*M* = 4.0, *SD* = .87) and talk to (*M* = 3.6, *SD* = .93) older adults. The majority of parents (88%) rated the quality of this contact with older adults as good or excellent.

In addition to their direct contact with older adults, parents also indicated which percent of the individuals living in their neighborhood were children or teens, young adults, middle-aged adults, and older adults. Overall, parents indicated that the largest part of their neighborhood consisted of middle-aged adults (*M* = 39%), followed by older adults (*M* = 28%), children and teens (*M* = 24%), and young adults (*M* = 19%).

Focusing on the percentage of older adults making up the participants’ neighborhood population, most parents (48%) indicated that older adults made up 20% to 30% of the neighborhood population. About a third (28%) of the parents reported that older

adults made up 50% to 70% of the neighborhood, and 7% of parents indicated that no older adults lived in their neighborhood.
